# Weight Loss Using an mHealth App Among Individuals With Obesity in Different Economic Regions of China: Cohort Study

**DOI:** 10.2196/48675

**Published:** 2024-01-16

**Authors:** Xinru Huang, Yefei Shi, Hongyun Yao, Mingjie Li, Zhijun Lei, Jiayun Shi, Bo Li, Weiwei Zhang, Weixia Jian

**Affiliations:** 1 Department of Endocrinology, Xinhua Hospital, Shanghai Jiaotong University School of Medicine, Shanghai, China; 2 Department of Cardiology, Shanghai Tenth People’s Hospital, Tongji University School of Medicine, Shanghai, China

**Keywords:** weight loss, obesity, normal-weight obesity, economic regions, mHealth app, mobile health, China, mHealth

## Abstract

**Background:**

With the increasing prevalence of obesity, weight loss has become a critical issue in China. Self-managed weight loss through a mobile health (mHealth) app may be a prospective method. However, its practicability in different economic regions of China is unknown.

**Objective:**

This study aims to evaluate the effectiveness of self-managed weight loss through an mHealth app among individuals with obesity in different economic regions of China and to demonstrate the feasibility of online self-management for weight loss.

**Methods:**

A total of 165,635 Chinese adults who signed up for the mHealth app were included to analyze the body composition characteristics of individuals from different economic regions by *χ*^2^ analyses. Furthermore, 2 types of participants with obesity using mHealth monitoring, including 74,611 participants with a BMI ≥24.0 kg/m^2^ and 22,903 participants with a normal BMI but an excessive percentage of body fat (PBF), were followed for 6 months to explore the weight loss and fat loss effects in different economic regions of China and to find independent predictors associated with weight loss success by 2-tailed Student *t* test and multivariable logistic regression analysis.

**Results:**

There were 32,129 users from low-income regions and 133,506 users from high-income regions. The proportion of users with obesity in low-income regions was higher than in high-income regions, both based on BMI (15,378/32,129, 47.9% vs 59,233/133,506, 44.4%; *P*<.001) and PBF classification (19,146/32,129, 59.6% vs 72,033/133,506, 54%; *P*<.001). Follow-up analyses showed that the weight loss effect among participants with overweight or obesity in low-income regions was greater than in high-income regions (mean –4.93, SD 6.41 vs mean –4.71, SD 6.14 kg; *P*<.001), while there was no significant difference in fat loss (mean –2.06%, SD 3.14% vs mean –2.04%, SD 3.19%; *P*=.54). In the population with normal-weight obesity, the weight loss (mean –2.42, SD 4.07 vs mean –2.23, SD 4.21 kg; *P*=.004) and fat loss effects (mean –1.43%, SD 2.73% vs mean –1.27%, SD 2.63%; *P*<.001) were stronger in high-income regions than in low-income regions. In addition, multivariable logistic regression analyses showed that age, baseline PBF, skeletal muscle rate, and measurement frequency were related to weight loss, whereas gender and baseline body metabolic rate only showed a correlation with weight loss in the population in high-income regions.

**Conclusions:**

This study found a high proportion of mHealth app users with obesity in low-income regions. Individuals with overweight and obesity in different economic regions of China experienced significant weight loss and fat loss using an mHealth app. Moreover, individuals in high-income regions paid more attention to body fat and had better fat reduction effects. Therefore, promoting self-monitoring of weight and PBF through an mHealth app could be an important intervention that could be implemented across all regions of China.

## Introduction

Obesity is a global health crisis that has reached pandemic proportions in many countries, including China [1,2]. The World Health Organization estimated that over 2.1 billion people worldwide are overweight or obese, and this number is expected to continue rising in the coming years [3,4]. Obesity is a significant risk factor for several chronic health conditions, including heart disease, stroke, diabetes, and certain types of cancer, and has been linked to decreased quality of life and premature death [5-7]. In China, the prevalence of overweight and obesity has substantially increased in recent years. This has been attributed to several factors, including increased urbanization, changes in dietary habits, and decreased physical activity levels [8,9]. The rising burden of obesity in China has led to growing concerns about the health and economic consequences of this trend and has prompted the need for effective weight loss.

Standard behavioral treatment for obesity included dietary and physical activity counseling and self-monitoring of body weight, activity, and diet [10,11]. Consistent self-weighing over time promoted the awareness of behaviors, environments, or situations that might lead to desired or undesired changes in weight. Researchers have established a correlation between self-weighing and successful weight loss, with studies showing that self-weighing significantly improved weight loss outcomes during the first 6 months of a weight loss intervention [12,13]. A new online weight management system, the smart body fat scale, calculates the percentage of body fat (PBF), records and synchronizes the data to mobile health (mHealth) apps, and offers more advantages than a traditional scale [14,15]. In addition to measuring PBF, smart body fat scales usually offer additional features such as the ability to track weight and body fat over time and the ability to measure other health-related metrics such as muscle mass [16]. The use of mHealth with smart body fat scales has become increasingly popular in recent years, as individuals seek to monitor their health and fitness more closely and make more informed decisions about their diets and physical activity patterns.

Currently, the increase in obesity rates is decelerating in high-income areas of China. In contrast, obesity is showing a significant increase in low-income areas, which indicates a requirement for targeted health policies to prevent a further increase in obesity among the general population [17]. Despite the growing popularity of smart body fat scales and the cost-effective potential of digital platforms for reaching a large number of individuals, there was limited research on their impact on weight loss across different economic regions. Here, we conducted a cohort study that analyzed the data of obesity-related anthropometric indices recorded through an mHealth app, which connected to the body fat scale, in individuals with overweight and obesity from low- and high-income regions of China. Our study aimed to investigate the weight loss effects of mHealth connected to smart body fat scales in different economic regions and to test our hypothesis that using mHealth in different economic regions could achieve significant weight loss and fat loss. This would also provide important insights into the potential for self-managed mHealth methods to promote healthy weight loss and improve health outcomes in this population.

## Methods

### Participants

**Figure 1.**
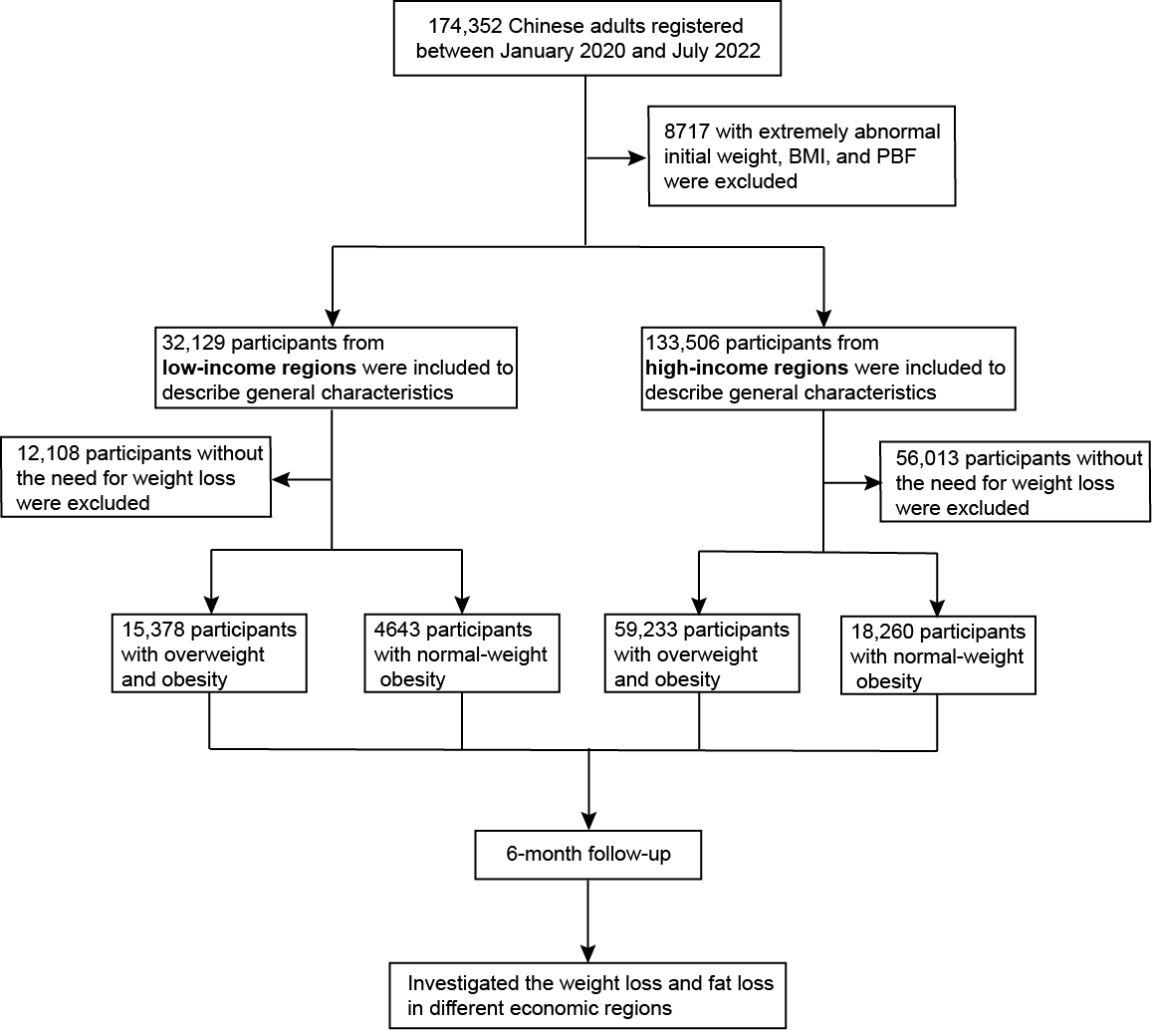
Study flowchart. PBF: percentage of body fat.

This study analyzed the data of 165,635 adults aged 18 to 79 years who signed up for the Qingniu Health app between January 2020 and July 2022 and lived in different economic regions of China. Users with a baseline BMI outside the 95% range were excluded. The baseline data were used to determine the general and body composition characteristics of mHealth users from different economic regions. Furthermore, we followed 74,611 participants with a BMI ≥24.0 kg/m^2^ and 22,903 participants with a normal BMI but an excessive PBF. All participants were followed for 6 months to assess weight and fat loss (Figure 1).

### Ethical Considerations

This study was approved by the Ethics Committee of Xinhua Hospitals (approval XHEC-D-2022-195). Electronic consent was provided by all participants. The data were deidentified, and our study did not involve compensation.

### Data Collection

The Qingniu Health app was used for the data collection. Participants were required to provide their baseline information, including gender, age, height, and city of residence, at the time of registering in the app. Body composition data, including weight, PBF, skeletal muscle rate (SMR), and basal metabolic rate (BMR), were measured using the same body fat scales (CS10C; Yolanda Technology Co., Ltd). These scales used multifrequency bioelectrical impedance analysis, as previously noted in another study [14].

### Subgroups

For the analyses, participants were divided into groups based on gender, age, BMI, PBF, measurement frequency, and economic class of the residential city. Participants younger than 40 years, 40-59 years of age, and 60 years and older were categorized as adults, middle-aged adults, and older adults, respectively. The BMI classification followed standards set by the Ministry of Health’s Disease Control Department for Chinese People. Those with a BMI less than 18.5 kg/m^2^ were considered underweight, those with a BMI between 18.5 and 23.9 kg/m^2^ were considered normal, those with a BMI between 24.0 and 27.9 kg/m^2^ were considered overweight, and those with a BMI of 28.0 kg/m^2^ or more were considered obese [9]. Male adults with a PBF ≥25.0% and female adults with a PBF ≥30.0% were considered obese [18], whereas the others were considered nonobese. Individuals with normal weight but had a high PBF were considered to have “normal-weight obesity.” Participants were also evenly divided into groups based on their frequency of measurements: low, medium, and high. First-tier, new first-tier, and second-tier cities were classified as high-income regions, whereas third-tier, fourth-tier, and fifth-tier cities were classified as low-income regions.

### Follow-Up Outcomes

The results of the follow-up were measured for 6 months (180±30 d) after the initial registration. A previous study showed that a 5% weight loss reduced the incidence of obesity-related diseases [19]. Therefore, participants who lost more than 5% of their initial body weight were considered to have achieved effective weight loss.

### Statistical Analyses

The continuous variables were reported as means and SDs, and the categorical variables were reported as counts and percentages. The frequency of measurements was characterized by medians and IQRs. Differences among groups were analyzed using the unpaired, 2-tailed Student *t* test, and the differences in the constituent ratios were evaluated with the *χ*^2^ test. Multivariable logistic regression analysis was used to identify the independent factors that influenced the results. The statistical analyses were performed using SPSS 26.0 (IBM Corp). A *P* value <.05 was considered statistically significant.

## Results

### General Characteristics of Participants

**Figure 2.**
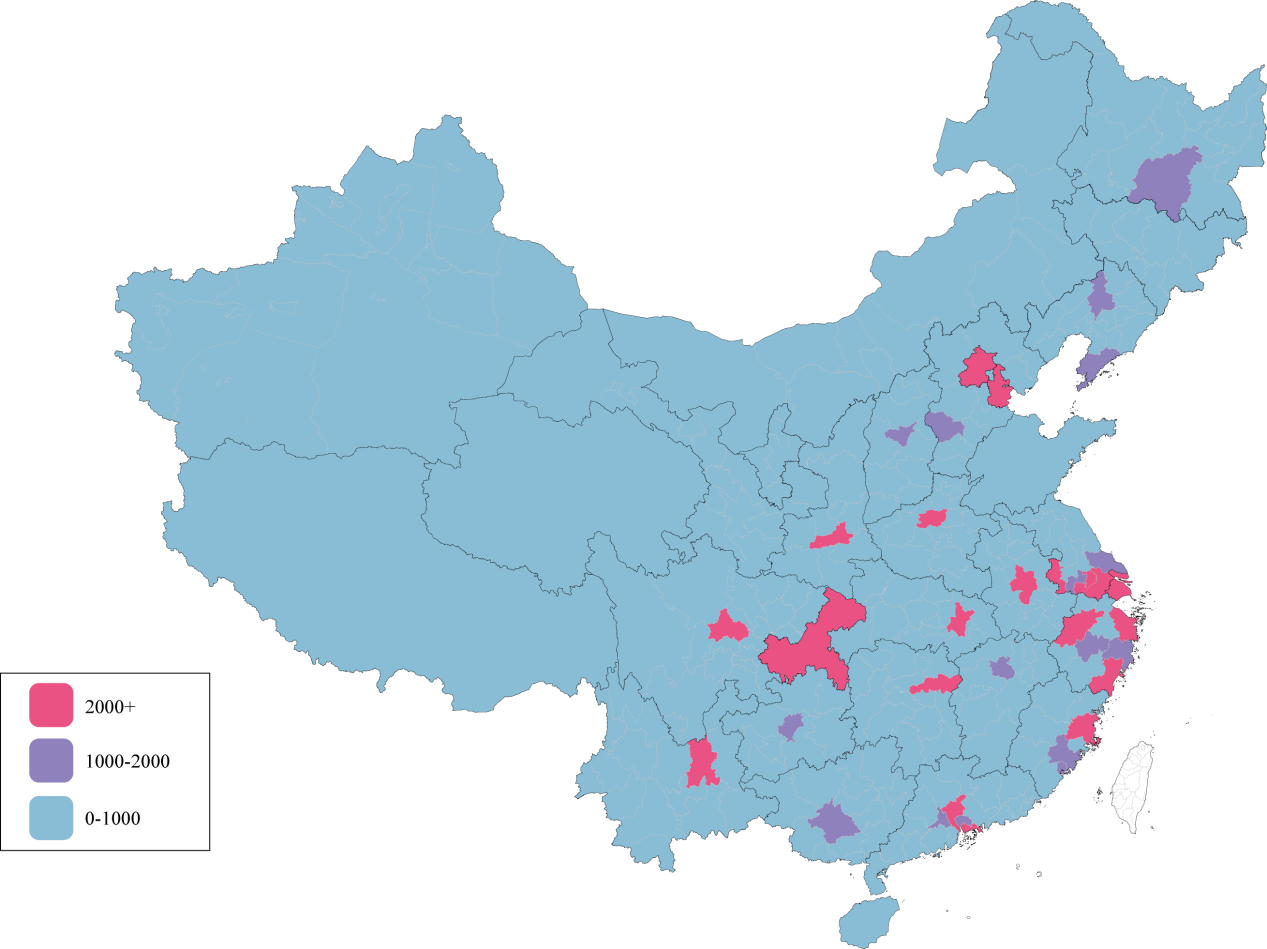
The number of users in mainland China. The pink areas have more than 2000 registrants, the purple areas have 1000-2000 registrants, and the blue areas have fewer than 1000 registrants.

A total of 165,635 Chinese adults registered in the Qingniu Health app were analyzed, consisting of 32,129 (19.4%) citizens from low-income regions and 133,506 (80.6%) citizens from high-income regions (Table 1). Users were mainly distributed in eastern coastal cities and inland provincial capitals (Figure 2). The majority of users were women (n=133,377, 80.5%) and those aged 18 to 40 years (n=126,796, 76.6%), and the proportion of women in low-income regions was greater than that in high-income regions (27,119/32,129, 84.4% vs 106,258/133,506, 79.6%; *P*<.001). Body composition analyses revealed that the proportion of individuals with overweight and obesity, as classified by BMI, and the proportion of individuals with obesity with a PBF above the upper limit were higher in low-income regions than in high-income regions (*P*<.001). On average, the baseline BMR and SMR of users in low-income regions were lower than those in high-income regions (BMR: mean 1339.99, SD 160.81 vs mean 1350.04, SD 174.93 kcal; *P*<.001; SMR: mean 41.33, SD 3.93 vs mean 42.06, SD 4.22%; *P*=.002). Based on the frequency of measurements during the 6 months, all users were equally divided into 3 groups: low (median 2.29, IQR 1.76 times), medium (median 7.26, IQR 3.95 times), and high (median 22.88, IQR 14.70 times).

**Table 1.** General characteristics of participants grouped by regional economic classification.

Characteristics		Low-income regions (n=32,129)	High-income regions (n=133,506)	*P* value^a^
Total participants (n=165,635), n (%)		32,129 (19.4)	133,506 (80.6)	N/A^b^
Women, n (%)		27,119 (84.4)	106,258 (79.6)	<.001
**Age group (years), n (%)**				<.001
	18-40	24,382 (75.9)	102,414 (76.7)	
	40-60	7419 (23.1)	29,438 (22)	
	60-80	328 (1)	1654 (1.2)	
**BMI (kg/m** ^**2**^ **), n (%)**				<.001
	Underweight	696 (2.2)	3662 (2.7)	
	Normal	16,055 (50)	70,611 (52.9)	
	Overweight	10,625 (33.1)	41,389 (31)	
	Obesity	4753 (14.8)	17,844 (13.4)	
**Percentage of body fat, n (%)**				<.001
	Normal	12,983 (40.4)	61,473 (46)	
	Obesity	19,146 (59.6)	72,033 (54)	
Baseline skeletal muscle rate (%), mean (SD)		41.33 (3.93)	42.06 (4.22)	.002
Baseline basal metabolic rate (kcal), mean (SD)		1339.99 (160.81)	1350.04 (174.93)	<.001
**Frequency of measurements,** **n (%)**				<.001
	Low	10,638 (33.1)	44,608 (33.4)	
	Medium	10,488 (32.6)	44,768 (33.5)	
	High	11,003 (34.2)	44,130 (33.1)	

a All *P* values were compared between different economic regions.

b N/A: not applicable.

### Baseline Characteristics of the Participants With Obesity

In our study, 2 types of obesity were defined based on BMI and PBF: overweight and obese with a BMI ≥24.0 kg/m^2^ and normal-weight obese with a normal BMI but an excessive PBF. A total of 74,611 participants with overweight and obesity and 22,903 participants with normal-weight obesity were included in the analysis of baseline characteristics. The proportion of users with overweight and obesity in low-income regions was close to half (15,378/32,129, 47.9%), rising to 62.3% (20,021/32,129) when users with normal-weight obesity were included. High-income regions (77,493/133,506, 58%) had a slightly lower proportion compared to low-income regions. There was no significant difference in the distribution of measurement frequency among individuals with overweight and obesity between different economic areas (*P*=.08). The percentage of middle-aged adults was higher (3958/15,378, 25.7% vs 14,748/59,233, 24.9%; *P*=.002) and the mean baseline BMR was lower in low-income areas compared to high-income areas (Table 2).

**Table 2.** Baseline characteristics of participants with obesity grouped by regional economic classification.

Characteristics		Overweight and obesity			Normal-weight obesity		
		Low-income regions (n=15,378)	High-income regions (n=59,233)	*P* value^a^	Low-income regions (n=4643)	High-income regions (n=18,260)	*P* value^a^
Total participants (low-income regions: n=32,129; high-income regions: n=133,506), n (%)		15,378 (47.9)	59,233 (44.4)	<.001	4643 (14.5)	18,260 (13.7)	<.001
Baseline basal metabolic rate (kcal), mean (SD)		1420.76 (165.97)	1446.57 (181.40)	<.001	1234.80 (77.04)	1236.48 (77.93)	.19
**Age group (years), n (%)**				.002			.001
	18-40	11,226 (73)	43,543 (73.5)		2541 (54.7)	10,024 (54.9)	
	40-60	3958 (25.7)	14,748 (24.9)		2018 (43.5)	7736 (42.4)	
	60-80	194 (1.3)	942 (1.6)		84 (1.8)	500 (2.7)	
**BMI (kg/m** ^**2**^ **), n (%)**				.06			N/A^b^
	Overweight	10,625 (69.1)	41,389 (69.9)		0 (0)	0 (0)	
	Obesity	4753 (30.9)	17,844 (30.1)		0 (0)	0 (0)	
**Percentage of body fat, n (%)**				<.001			N/A
	Normal	875 (5.7)	5462 (9.2)		0 (0)	0 (0)	
	Obesity	14,503 (94.3)	53,771 (90.8)		4643 (100)	18,260 (100)	
**Frequency of measurements, n (%)**				.08			.06
	Low	4308 (28)	16,779 (28.3)		1342 (28.9)	4996 (27.4)	
	Medium	4850 (31.5)	19,073 (32.2)		1491 (32.1)	6134 (33.6)	
	High	6220 (40.4)	23,381 (39.5)		1810 (39)	7130 (39)	

a All *P* values were compared between different economic regions.

b N/A: not applicable.

Moreover, the proportion of individuals with normal-weight obesity was also slightly higher in low-income regions than in high-income regions (4643/32,129, 14.5% vs 18,260/133,506, 13.7%; *P*<.001), and there was no significant difference in the mean baseline BMR of individuals with normal-weight obesity (mean 1234.80, SD 77.04 vs mean 1236.48, SD 77.93 kcal; *P*=.19; Table 2). Compared with the percentage of middle-aged adults with overweight and obesity (defined by BMI; 18,748/74,611, 25.1%), the percentage of middle-aged adults with normal-weight obesity was higher (9754/22,903, 42.6%).

### Weight Loss and Fat Loss of the Participants With Obesity

We followed the aforementioned individuals with obesity for 6 months and found that participants with overweight and obesity who used the self-monitoring mHealth app experienced significant weight loss and fat loss in both regions (low-income regions: mean –4.93, SD 6.41 kg; high-income regions: mean –4.71, SD 6.14 kg; *P*<.001). Men with overweight and obesity in low-income regions lost weight markedly more than those in high-income regions (*P*=.01; Table 3). When grouped by age, baseline BMI, and measurement frequency, weight loss was greater among users in low-income regions than in high-income regions. Additionally, individuals with normal-weight obesity showed less weight loss than individuals with overweight and obesity. In contrast, in the population with normal-weight obesity, individuals lost more weight in high-income regions than those in low-income regions.

**Table 3.** Weight loss grouped by regional economic classification.

Characteristics		Weight loss in the overweight and obesity group (kg), mean (SD)			Weight loss in the normal-weight obesity group (kg), mean (SD)		
		Low-income regions	High-income regions	*P* value^a^	Low-income regions	High-income regions	*P* value^a^
All participants		–4.93 (6.41)	–4.71 (6.14)	<.001	–2.23 (4.21)	–2.42 (4.07)	.004
**Gender**							
	Men	–4.40 (6.81)	–4.08 (6.51)	.01	–1.62 (9.31)	–1.72 (4.55)	.91
	Women	–5.08 (6.27)	–4.99 (5.94)	.11	–2.24 (4.14)	–2.43 (4.06)	.003
**Age group (years)**							
	18-40	–5.08 (6.68)	–4.82 (6.37)	<.001	–2.51 (4.39)	–2.72 (4.30)	.03
	40-60	–4.56 (5.59)	–4.39 (5.39)	.07	–1.91 (3.95)	–2.11 (3.68)	.03
	60-80	–3.68 (4.86)	–4.33 (5.75)	.14	–1.30 (4.10)	–1.31 (4.55)	.98
**BMI (kg/m** ^**2**^ **)**							
	Overweight	–4.07 (5.48)	–3.92 (5.16)	.01	N/A^b^	N/A	N/A
	Obesity	–6.85 (7.77)	–6.53 (7.64)	.01	N/A	N/A	N/A
**Percentage of body fat (%)**							
	Obesity	–5.08 (6.40)	–4.94 (6.19)	.01	–2.23 (4.21)	–2.42 (4.07)	.004
**Frequency of measurements**							
	Low	–3.24 (7.44)	–2.92 (6.74)	.009	–0.84 (5.00)	–1.28 (4.71)	.003
	Medium	–3.96 (5.72)	–3.84 (5.53)	.20	–1.94 (3.61)	–2.09 (3.72)	.18
	High	–6.86 (5.58)	–6.70 (5.57)	.046	–3.50 (3.64)	–3.51 (3.57)	.88

a All *P* values were compared between different economic regions.

b N/A: not applicable.

Considering that the goal of participants with normal-weight obesity was to reduce body fat, we further explored the differences in the efficiency of fat loss in different economic regions. We found that the fat loss effect on the population with normal-weight obesity was greater in high-income regions. However, grouped by gender, age, and measurement frequency, we conducted unpaired 2-tailed Student *t* tests for each group and found that there were no differences in the fat loss effect on people with overweight and obesity in different economic regions (Table 4).

**Table 4.** Fat loss of participants with obesity grouped by regional economic classification.

Characteristics		Fat loss in the overweight and obesity group (PBF^a^; %), mean (SD)			Fat loss in the normal-weight obesity group (PBF; %), mean (SD)		
		Low-income regions	High-income regions	*P* value^b^	Low-income regions	High-income regions	*P* value^b^
All participants		–2.06 (3.14)	–2.04 (3.19)	.54	–1.27 (2.63)	–1.43 (2.73)	<.001
**Gender**							
	Men	–2.00 (3.57)	–2.03 (3.76)	.73	–5.13 (8.94)	–4.61 (7.39)	.67
	Women	–2.07 (3.01)	–2.04 (2.91)	.35	–1.23 (2.46)	–1.38 (2.55)	<.001
**Age group (years)**							
	18-40	–2.13 (3.26)	–2.11 (3.30)	.48	–1.47 (2.99)	–1.66 (3.01)	.004
	40-60	–1.87 (2.82)	–1.85 (2.86)	.67	–1.04 (2.02)	–1.18 (2.32)	.01
	60-80	–1.31 (2.12)	–1.70 (2.83)	.07	–0.90 (3.29)	–0.81 (2.52)	.77
**BMI (kg/m** ^**2**^ **)**							
	Overweight	–1.85 (2.92)	–1.82 (2.95)	.38	N/A^c^	N/A	N/A
	Obesity	–2.52 (3.55)	–2.55 (3.65)	.67	N/A	N/A	N/A
**PBF (%)**							
	Obesity	–2.13 (3.09)	–2.15 (3.13)	.54	–1.27 (2.63)	–1.43 (2.73)	<.001
**Frequency of measurements**							
	Low	–1.42 (3.80)	–1.33 (3.60)	.13	–0.70 (3.24)	–0.91 (3.12)	.03
	Medium	–1.64 (2.92)	–1.68 (2.94)	.46	–0.98 (1.98)	–1.23 (2.57)	<.001
	High	–2.81 (2.61)	–2.84 (2.90)	.52	–1.93 (2.44)	–1.98 (2.48)	.50

a PBF: percentage of body fat.

b All *P* values were compared between different economic regions.

c N/A: not applicable.

### Independent Factors Linked With Successful Weight Loss in Different Economic Regions

We conducted multivariable logistic regression analyses to examine the relationship between various baseline factors and successful weight loss in individuals with overweight and obesity. In the population from low-income regions, younger participants with higher baseline PBF, baseline SMR, and measurement frequency were more likely to succeed in weight loss (Table 5). In addition, women in high-income regions were more likely to achieve successful weight loss. The results indicated that the frequency of measurement was the most critical independent factor in both low-income (odds ratio 5.036, 95% CI 4.618-5.491; *P*<.001) and high-income (odds ratio 5.271, 95% CI 5.042-5.511; *P*<.001) regions, particularly a high measurement frequency. Similarly, we further estimated the dependent factors associated with successful fat loss in the populations with overweight and obesity. In different economic regions, younger men with a higher baseline PBF and measurement frequency and lower BMR were more likely to lose body fat (Table 5).

**Table 5.** Factors linked with successful weight loss and fat loss in overweight and obese group.

Characteristics		Weight loss				Fat loss			
		Low-income regions		High-income regions		Low-income regions		High-income regions	
		OR^a^ (95% CI)	*P* value	OR (95% CI)	*P* value	OR (95% CI)	*P* value	OR (95% CI)	*P* value
Gender		N/A^b^	N/A	0.748 (0.680-0.823)	<.001	2.568 (2.139-3.081)	<.001	3.561 (3.211-3.949)	<.001
Age		0.981 (0.977-0.985)	<.001	0.983 (0.981-0.985)	<.001	0.971 (0.967-0.975)	<.001	0.972 (0.970-0.974)	<.001
Baseline PBF^c^		1.109 (1.087-1.132)	<.001	1.079 (1.071-1.088)	<.001	1.047 (1.034-1.060)	<.001	1.011 (1.003-1.019)	.007
Baseline SMR^d^		1.052 (1.029-1.075)	<.001	1.036 (1.025-1.048)	<.001	N/A	N/A	0.937 (0.925-0.948)	<.001
Baseline BMR^e^		N/A	N/A	1.001 (1.000-1.001)	<.001	0.999 (0.999-1.000)	<.001	0.999 (0.999-0.999)	<.001
**Frequency of measurements classification **									
	Medium-low	1.611 (1.479-1.755)	<.001	1.771 (1.695-1.851)	<.001	1.557 (1.427-1.700)	<.001	<0	<.001
	High-low	5.036 (4.618-5.491)	<.001	5.271 (5.042-5.511)	<.001	4.606 (4.224-5.023)	<.001	N/A	<.001

a OR: odds ratio.

b N/A: not applicable.

c PBF: percentage of body fat.

d SMR: skeletal muscle rate.

e BMR: basal metabolic rate.

## Discussion

### Principal Findings

After 6 months of follow-up, the results showed that significant weight and fat loss were found in participants with overweight and obesity using the mHealth app in different economic regions. Furthermore, individuals in low-income regions lost more weight than individuals in high-income regions in the population with overweight and obesity, and there was no difference between individuals in fat loss. Providing further health education and online weight loss monitoring in low-income regions was beneficial to the population. Interestingly, in the population with normal-weight obesity, individuals in high-income regions lost more fat than individuals in low-income regions.

### Comparison With Prior Work

Obesity has become a major public health challenge worldwide, with strong links to metabolic disorders such as cardiovascular diseases [5] and diabetes [20]. With the transformation of China’s economic and social structure, dietary patterns and nutritional status have undergone significant changes, and the problem of obesity has become increasingly serious [21]. Addressing obesity can help reduce the incidence of chronic diseases [22], and the economic benefits of long-term nonsurgical weight loss in individuals with obesity have been well established [23]. In the previous study, we found that a large number of Chinese individuals with overweight and obesity were able to achieve weight loss goals through an mHealth app during a long-term follow-up, leading us to conclude that mHealth with body fat scales might be a promising method for weight loss and fitness [24]. However, given the vast territory and large population of China (Figure 2), we aimed to investigate whether self-management through mHealth could achieve consistent results across different economic regions, further demonstrating the feasibility of online self-management for weight loss.

The mHealth app with the body fat scale is a widely used self-weighing tool due to its affordability and convenience, different from the traditional paper record [25]. We analyzed the user data of the Qingniu Health app linked to the body fat scale. We divided all the included users into 2 groups: high-income regions and low-income regions according to the economic classification of their living regions. Results showed that the number of users in high-income regions was more than quadruple that in low-income regions (n=133,506 vs n=32,129), and the vast majority of users were women in both groups, which was similar to other research [26]. This revealed that people in high-income regions paid more attention to obesity prevention and monitoring, particularly women. Furthermore, the age distribution of users showed a majority of young and middle-aged people. When grouped by baseline BMI and PBF, both the proportion of people with an excessive BMI and the proportion of people with an excessive PBF in low-income regions were significantly higher than those in high-income regions, as noted in a comment published in *Nature* [27]. In all high-income countries, overweight and obesity levels were already higher in rural areas than in urban areas, and the same phenomenon might be occurring in China [27,28]. These results suggest that self-management and lifestyle interventions are required to prevent further development of metabolic-related diseases in people with excess body weight and body fat in low-income areas.

Previous studies have established a close association between socioeconomic status and the risk of obesity [29-31]. Despite this, the relationship between weight loss and economic regions among Chinese adults remains largely unknown. Our study of the user data of the Qingniu Health app linked to the body fat scale found that nearly half of the users met the criteria for a diagnosis of obesity based on their BMI. The average baseline BMR for users in low-income regions was lower, and the individual BMR was correlated with multiple factors such as age, gender, body composition, and BMI [32]. There were several users with overweight and obesity with a normal baseline PBF and some people with a normal BMI but excessive PBF, which we called the population with normal-weight obesity. Normal-weight obesity was closely associated with metabolic and cardiovascular diseases [33]. Our findings showed that the proportion of the population with normal-weight obesity in low-income regions was higher than in high-income regions. Moreover, the increase in the proportion of middle-aged adults with normal-weight obesity was associated with an increase in biological age, a lack of physical activity, and other factors. This phenomenon highlighted the need for increased focus on body fat control in this age group [34]. As expected, we found no statistical difference in the composition of measurement frequency between participants in different economic regions, showing that users were able to maintain a certain frequency of use with the app, which suggested that promoting the use of mHealth in our country was a feasible and effective approach to monitoring obesity.

In our previous study [24], we found that a younger age was the most important contributing factor to fat loss success. In this study, the age composition of participants with normal-weight obesity in high-income regions was not different from that in low-income regions but showed a more obvious trend of weight loss and fat loss, reflecting that participants from high-income regions had focused on body fat in addition to body weight. To better understand the factors affecting successful weight loss in different economic regions, we found that age, baseline PBF, SMR, and measurement frequency were significant predictors for weight loss success, whereas gender and baseline BMR only showed a correlation with weight loss in the population in high-income regions, which suggests women in high-income regions paid more attention to self-management than those in low-income regions.

### Limitations

Our study had a large sample size and a prolonged follow-up period, which accurately reflected the real-world situation of online self-management in China. Nevertheless, there were certain limitations in our study. We must acknowledge that our study was a nonrandomized cohort study without controls. Nevertheless, we observed a significant weight loss outcome in individuals with overweight and obesity from the 2 different economic regions in this large-scale follow-up study. Furthermore, individuals who used the smart body fat scale with a higher frequency exhibited better weight loss results compared to those with a lower use frequency. This suggests that the use of an mHealth app connected to a smart body fat scale has a certain impact on effective weight reduction, regardless of economic region. In the future, we can consider adding control groups using alternative weight loss methods to further clarify the role of using an mHealth app connected to a smart body fat scale in self-initiated weight and fat loss. Additionally, we did not consider the 2 important influencing factors of participants’ diet and exercise volume. We also did not consider the overall health status of the participants; factors such as hydration level might impact the accuracy of body fat measurement using bioelectrical impedance analysis.

### Conclusions

A high proportion of individuals with obesity from low-income regions was found in our study, and individuals with overweight and obesity who used body fat scales in different economic regions of China experienced significant weight loss and fat loss. Individuals from high-income regions paid more attention to body fat and had better fat loss than those from low-income regions, and in the middle-aged population, the issue of normal-weight obesity required more attention. Therefore, promoting self-monitoring of weight and fat through the use of body fat scales connected to an mHealth app could be an important intervention measure for the population with overweight and obesity across all regions of China.

## Abbreviations

BMR: basal metabolic rate
mHealth: mobile health
PBF: percentage of body fat
SMR: skeletal muscle rate
